# Variation at the Calpain 3 gene is associated with meat tenderness in zebu and composite breeds of cattle

**DOI:** 10.1186/1471-2156-9-41

**Published:** 2008-07-01

**Authors:** William Barendse, Blair E Harrison, Rowan J Bunch, Merle B Thomas

**Affiliations:** 1 CSIRO Livestock Industries and CRC for Cattle and Beef Quality, Queensland Bioscience Precinct, 306 Carmody Road, St. Lucia 4067, Australia

## Abstract

**Background:**

Quantitative Trait Loci (QTL) affecting meat tenderness have been reported on Bovine chromosome 10. Here we examine variation at the Calpain 3 (*CAPN3*) gene in cattle, a gene located within the confidence interval of the QTL, and which is a positional candidate gene based on the biochemical activity of the protein.

**Results:**

We identified single nucleotide polymorphisms (SNP) in the genomic sequence of the *CAPN3 *gene and tested three of these in a sample of 2189 cattle. Of the three SNP genotyped, the *CAPN3:c.1538+225G>T *had the largest significant additive effect, with an allele substitution effect in the Brahman of *α *= -0.144 kg, SE = 0.060, *P *= 0.016, and the polymorphism explained 1.7% of the residual phenotypic variance in that sample of the breed. Significant haplotype substitution effects were found for all three breeds, the Brahman, the Belmont Red, and the Santa Gertrudis. For the common haplotype, the haplotype substitution effect in the Brahman was *α *= 0.169 kg, SE = 0.056, *P *= 0.003. The effect of this gene was compared to Calpastatin in the same sample. The SNP show negligible frequencies in taurine breeds and low to moderate minor allele frequencies in zebu or composite animals.

**Conclusion:**

These associations confirm the location of a QTL for meat tenderness in this region of bovine chromosome 10. SNP in or near this gene may be responsible for part of the overall difference between taurine and zebu breeds in meat tenderness, and the greater variability in meat tenderness found in zebu and composite breeds. The evidence provided so far suggests that none of these tested SNP are causative mutations.

## Background

The status of DNA tests for meat tenderness has been recently discussed [1] and so far there are only two genes identified that have consistent effects on meat tenderness reported in the literature, that for Calpastatin and Calpain 1 [2-5]. There are two polymorphisms in Calpain 1 (*CAPN1*), one appearing to be more useful in taurine breeds and one more useful in zebu breeds. On the other hand, although several possible causative mutations have been identified in Calpastatin (*CAST*), variation at this gene appears to affect all breed types.

Quantitative trait loci (QTL) for meat tenderness were located to bovine chromosome 10 in a Charolais × Brahman experimental population [1]. The authors suggested that the Calpain 3 (*CAPN3*) gene could be implicated because it occurred within the confidence interval of the location of the QTL and that calpain 3 protein activity had been implicated in meat tenderness in sheep [6,7], although DNA variants for *CAPN3 *have not been tested to determine whether they are associated with meat tenderness in any species.

Here we report the identification of single nucleotide polymorphisms (SNP) in *CAPN3 *and the testing of these SNP for associations to meat tenderness in a large sample of cattle of several breeds. We found that polymorphisms in the gene were not common in taurine cattle, so associations were only tested in zebu and composite animals, the term crossbred being reserved for animals with parents from different breeds. Zebu breeds and their tropically adapted composites are reported to have a 2 to 3 times greater heritability for meat tenderness than taurine breeds as well as a slightly higher mean value for shear force, a measure of meat tenderness [8]. These SNP appear to contribute primarily to the variation in tenderness in zebu or tropically adapted composite cattle.

## Methods

### Cattle Samples

The Beef CRC cattle, the methods of measurement of phenotypes, and the methods of DNA extraction have been reported previously [3,9,10]. After initial testing (cf. Results) the study was performed on the Brahman (BRM), Belmont Red (BEL) and Santa Gertrudis (SGT) animals in the sample. For the sample that was used, the number of sires and average number of offspring per sire for Brahman was 59 sires and a mean of 10.7 offspring per sire, for Belmont Red was 69 sires and a mean of 12.8 offspring per sire, and for Santa Gertrudis was 72 sires and a mean of 9.2 offspring per sire.

Meat tenderness was measured in kilograms using peak force measurements for the *Musculus longissimus lumborum *(LLPF) as described previously [9]. The least square means, standard deviations and heritabilities of this trait have been previously published for these animals [11]. The LLPF measurements of all of these animals were analysed by a general linear mixed model using restricted maximum likelihood in ASReml [12] where LLPF ~*N*(μ + kill group + herd of origin + age of slaughter + carcass weight + sire, σ^2^_e_) and where sire was fitted as a random effect as previously described [3,13], and variance components were estimated. The model accounted for the fixed effects of herd of origin and kill group, for the covariates of age at slaughter and carcass weight and the random effect of sire. There are several herds of origin associated with each breed so the average genetic effects due to breed and sire were included in the model as well as fixed environmental treatment effects; this avoids false associations due to inappropriate lumping of groups. To prevent the model being affected by the particular choice of animals or the estimates of the allelic effects being influenced by missing genotypic data, phenotypes for all animals were included in the model rather than only those that were genotyped.

#### SNP discovery

Genomic DNA for SNP discovery was obtained by direct sequencing of PCR products. Primers were obtained from the cDNA sequence for *CAPN3 *deposited by Ilian and coworkers (Genbank AF115744.1). The location of the exons was placed onto the sequence using a comparative analysis of sequences from other species. Then primers to amplify three segments of the gene were synthesized, one set flanking exon 6, one incorporating introns 10 and 11 and one that incorporated from intron 24 towards the 3' UTR (untranslated region) of the gene. DNA sequences were obtained using the Big-Dye 3.1 terminator kit from Applied Biosystems Inc. (ABI, Foster City, CA), using the manufacturer's instructions. Each fragment was sequenced in both directions in 10 animals including the Angus, Shorthorn and Brahman breeds. The DNA sequences were compared to sequences in Genbank using BLAST [14] to confirm the identity of the exons. These sequences were assessed, then they were assembled into contigs using phred and Phrap [15] and viewed using Consed [16]. PolyPhred [17] was used to identify variable bases with a threshold phred quality score (PQS) of 20. SNP were described using standard nomenclature [18].

#### SNP genotyping

**Figure 1:**
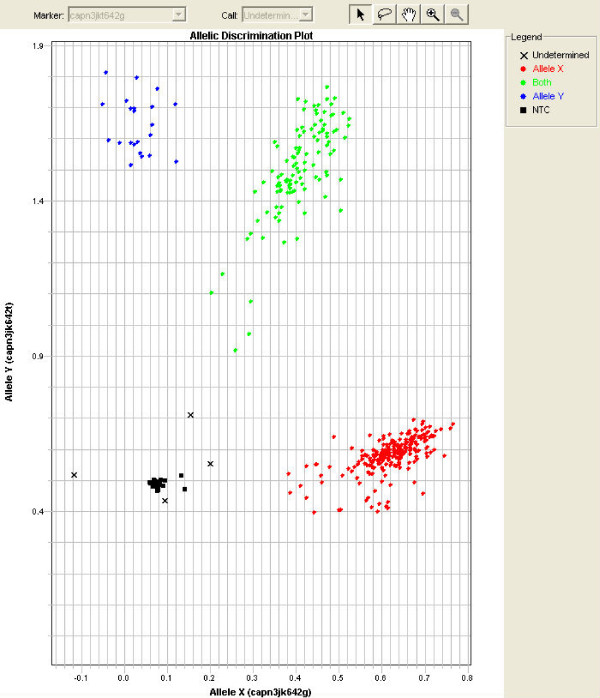
The Taqman™ assay for *CAPN3:c.1538+225G>T *on cattle DNA.

SNP were genotyped using the *Taqman*™ MGB allele discrimination method (ABI, Foster City, CA) as before [3] by two individuals. An example of one of the SNP is shown in Figure 1 following the recommendation of the NCI-NHGRI working group on initial reports of associations of genotypes and phenotypes [19]. Scoring was always performed without knowing the phenotypes. For ease of analysis and compact reporting of the data in the tables, genotypes were coded as 0, 1, 2 and 5 where 5 is unknown, 1 is always the heterozygote, 0 is the homozygote higher up the alphabet and 2 is the homozygote lower down the alphabet – so CC is 2 when AA is the alternative homozygote but 0 when GG is the alternative homozygote.

Genotypes were tested for Hardy-Weinberg Equilibrium (HWE), inferred haplotypes were used to calculate linkage disequilibrium (LD) as measured by *r*^2 ^and differences in frequency between breeds were tested as previously described [3,20,21]. The significance of an *r*^2 ^value was determined as *χ*^2^_1 _= N*r*^2 ^where N is the sample size [20]. The genotypes and residual meat tenderness values were used to estimate the additive and allele substitution effects and their standard errors by regressing the number of copies of an allele against the trait value using the equation α = *a *+ *d*(*q*-*p*) where α is the allele substitution effect, *a *is the additive effect, *d *is the dominance effect, and *p *+ *q *= 1 and are the allele frequencies [3,22-25]. Equivalent results for this regression can be obtained from a linear model in a statistical program, treating the genotypes as a variate of 0, 1 and 2 copies of a particular allele. To obtain breed specific estimates the genotypes are nested within breed. The probability of the association was obtained directly from the t-test of the effect divided by its standard error. The proportion of the residual variance explained by a SNP or combination of SNP was calculated as the square of the correlation between the genotypes and the residual trait values. Epistasis between *CAPN3 *and *CAST *SNP was tested using the software EPEE [3], which uses the G2A model [22] to assess epistasis between two loci.

To estimate the effect of *CAPN3 *haplotypes on meat tenderness, the number of copies of the common haplotype was regressed against the residual meat tenderness values in the same way as the allele substitution effect. Two-locus haplotypes were used in the analysis for the following reasons. Firstly, two-locus SNP haplotypes can be obtained unambiguously from genotypes, except for the double heterozygote and it was ignored in this analysis. Secondly, the analysis was to regress the trait against the number of copies of the common haplotype. Using two-locus haplotypes reduces the genotypic heterogeneity of the one-copy and the no-copy groups, compared to analyses that use haplotypes based on more SNP. The expected number of genotypes equals 3^n ^for n loci. Thirdly, the larger the number of loci making up the haplotype the smaller the number of individuals in the two-copies group, which will affect the accuracy of the estimates. The additive effect and the haplotype substitution effect of the common haplotype were calculated in the same way as the additive and allele substitution effects (cf. above). The use of two-locus haplotypes has the advantage that the analysis is essentially of the interval between the two SNP. With a large number of SNP one might restrict the analysis to adjacent intervals, but as there are only three SNP and therefore only three possible comparisons, all three intervals were analysed.

## Results

We examined the DNA sequence traces to find polymorphisms that were present in all breeds. All of the SNP that were discovered were either polymorphic in the Brahman animals in the panel or were differences between Brahman on the one hand and the Angus and Shorthorn on the other hand. We chose three SNP that were polymorphic in the Brahman breed for further study, one for each of the fragments that we had sequenced (Table 1). These were 1) the amino acid replacing *CAPN3:c.53T>G *(Met18Arg), dbSNP id ss102661473, 2) the intronic *CAPN3:c.1538+225G>T*, ss102661474, and 3) the intronic *CAPN3:c.2443-103G>C*, ss102661475.

**Table 1:** Primer sequences for the *CAPN3 *single nucleotide polymorphisms

SNP	Name	5'-3' sequence
*CAPN3:c.53T>G*		
MGB-probes	CAPN3E6100T	6FAM-TGAGCCCATGTCC
	CAPN3E6100G	VIC-TGAGCCCAGGTCC
Primers	CAPN3E6G100TU1	ATGCCGACCGTCATTAGC
	CAPN3E6G100TD1	GAGTAGATGCCACTTGGGTTTC
*CAPN3:c.1538+225G>T*		
MGB-probes	CAPN3JK-V2-642G	VIC-TACACGCTCACATGC
	CAPN3JK-V2-642T	6FAM-ACACTCTCACATGCT
Primers	CAPN3JKG642TU2	ATTGCATGGCCTCCTGAC
	CAPN3JKG642TD2	CTCCAGAACACCTCTGGACTG
*CAPN3:c.2443-103G>C*		
MGB-probes	CAPN3X3-266G	6FAM-CTTCCGTGTCTGGC
	CAPN3X3-266C	VIC-TCTTCCCTGTCTGGC
Primers	CAPN3X3G266CU2	AGCCTCAGTTATGGCTTTATGC
	CAPN3X3G266CD1	AGATCAGAATGCACAATGAGACA

To determine whether it was worth genotyping animals of all breeds, because these SNP were discovered in Brahman animals, we tested a group of taurine animals of three breeds. Testing the first SNP, *c.2443-103G>C*, there was 1 heterozygote in 124 taurine animals from the Angus, Hereford and Shorthorn breeds, so we did not use taurine samples for further genotyping because the chance of finding sufficient homozygotes of each genotype in those breeds would be low.

We examined the genotypes for deviations from HWE and for differences between breeds because significant differences in these measures could affect the subsequent analysis of association between DNA markers and the trait. The genotypes for all three SNP were in HWE and there were highly significant differences in genotype frequencies between breeds. The allele frequencies of the minor allele in the composite breeds were approximately half that found in the Brahman breed (Table 2), and given the very large sample sizes, these differences were extremely significant.

**Table 2:** The means and standard errors for *CAPN3 *and *CAST *SNP and peak force in the longissimus muscle

Locus	Breed	Homozygote 0			Heterozygote 1			Homozygote 2		
		N^1^	x^2^	SE^3^	N	x	SE	N	x	SE
*CAPN3*										
		GG			GT			TT		
*c*.53*T*>*G*	BRM^4^	62	-0.02	0.10	238	-0.11	0.05	314	0.08	0.05
*c*.53*T*>*G*	BEL^5^	26	0.06	0.12	203	0.10	0.05	609	-0.02	0.03
*c*.53*T*>*G*	SGT^6^	22	0.05	0.14	203	-0.06	0.05	405	0.04	0.04
		GG			GT			TT		
*c.1538+225G>T*	BRM	153	-0.10	0.06	279	-0.02	0.05	134	0.19	0.08
*c.1538+225G>T*	BEL	673	0.02	0.03	113	-0.05	0.07	3	0.79	0.82
*c.1538+225G>T*	SGT	371	0.04	0.04	213	-0.00	0.05	32	-0.18	0.08
		CC			CG			GG		
*c.2443-103G>C*	BRM	58	-0.06	0.10	209	-0.10	0.05	295	0.08	0.05
*c.2443-103G>C*	BEL	19	0.15	0.15	154	0.11	0.06	487	-0.02	0.03
*c.2443-103G>C*	SGT	19	0.03	0.13	134	-0.05	0.06	393	0.04	0.04
*CAST*										
		AA			AG			GG		
*c.2832A>G*	BRM	172	-0.12	0.06	279	-0.00	0.05	105	0.11	0.08
*c.2832A>G*	BEL	379	-0.06	0.04	239	0.16	0.05	28	0.18	0.16
*c.2832A>G*	SGT	256	-0.07	0.05	208	0.10	0.06	47	0.17	0.11

^1^Joint genotypes and phenotypes for genotype 0, ^2 ^mean residual phenotype for genotype 0, ^3 ^Standard error for the mean phenotype of genotype 0, ^4 ^BRM Brahman, ^5 ^BEL Belmont Red, ^6 ^SGT Santa Gertrudis

To determine whether there may be high levels of LD between alleles, which may affect the interpretation of the allelic association to the trait, *r*^2 ^was calculated from haplotypes inferred using the EM algorithm. The alleles were in significant LD in all breeds (Table 3). Some of the *r*^2 ^values were small (*r*^2^~0.01) and were significant only because the sample size was large. *CAPN3:c.1538+225G>T *showed moderate LD to *c.53T>G *and *c.2443-103G>C *in Brahman but very low LD in Belmont Red and Santa Gertrudis. *CAPN3:c.53T>C *and *c.2443-103G>C *showed very high LD in all breeds. All the *CAPN3 *SNP showed very low LD to *CAST:c.2832G>A *in all breeds with an average *r*^2 ^= 0.003, SE = 0.001, N = 9, as expected for unlinked loci. None of the *CAPN3-CAST r*^2 ^values were significant.

**Table 3:** Estimated LD between *CAPN3 *alleles in different breeds

SNP pair	Breed	*r*^2^	P
*c.53T>G c.1538+225G>T*	BRM	0.366	<<0.00001
*c.53T>G c.1538+225G>T*	BEL	0.008	0.01513
*c.53T>G c.1538+225G>T*	SGT	0.062	<<0.00001
*c.53T>G c.2443-103G>C*	BRM	0.915	<<0.00001
*c.53T>G c.2443-103G>C*	BEL	0.853	<<0.00001
*c.53T>G c.2443-103G>C*	SGT	0.801	<<0.00001
*c.1538+225G>T c.2443-103G>C*	BRM	0.374	<<0.00001
*c.1538+225G>T c.2443-103G>C*	BEL	0.014	0.00317
*c.1538+225G>T c.2443-103G>C*	SGT	0.040	<<0.00001

BRM, Brahman, BEL, Belmont Red, SGT, Santa Gertrudis

Significant associations were found between a *CAPN3 *SNP and LLPF in the Brahman and Belmont Red breeds but not in the Santa Gertrudis (Table 4). The strongest effect, in terms of residual variance explained, was found in the Brahman breed for the *c.1538+225G>T *SNP, where the *GG *genotype had increased meat tenderness. Both additive and allele substitution effects (α = 0.19 σ_p_), were significant and the dominance effect was not significant. Although the Belmont Red breed also showed a large significant additive effect, this is based on a few animals and so should be treated with caution. In particular, the allele substitution effect was not significant and was essentially zero. The Belmont Red breed showed significant allele substitution effects for both *c.53T>G *and *c.2443-103G>C*. The Brahman breed showed large but non-significant effects for these latter two SNP, but the favourable genotype was reversed and so the allele substitution effect was of opposite polarity.

**Table 4:** Association between the *CAPN3 *and *CAST *SNP and peak force in the longissimus muscle

Locus	Breed	p_0_^1^	V_r_^2^	*a*^3^kg	SE_a_^4^	P_*a*_^5^	α^6^kg	SE_α_	P_α_
*CAPN3*									
*c*.53*T*>*G*	BRM^7^	0.29	0.0111	-0.046	0.082	0.578	-0.103	0.060	0.088
*c*.53*T*>*G*	BEL^8^	0.15	0.0043	0.037	0.057	0.520	0.089	0.044	0.046
*c*.53*T*>*G*	SGT^9^	0.20	0.0038	0.005	0.074	0.942	-0.058	0.058	0.315
*c.1538+225G>T*	BRM	0.52	0.0172	-0.147	0.066	0.026	-0.144	0.060	0.016
*c.1538+225G>T*	BEL	0.92	0.0052	-0.380	0.046	0.000†	0.003	0.037	0.944
*c.1538+225G>T*	SGT	0.78	0.0040	0.108	0.066	0.102	0.068	0.060	0.260
*c.2443-103G>C*	BRM	0.29	0.0108	-0.067	0.080	0.402	-0.115	0.063	0.068
*c.2443-103G>C*	BEL	0.15	0.0061	0.083	0.065	0.203	0.114	0.053	0.031
*c.2443-103G>C*	SGT	0.16	0.0021	-0.005	0.087	0.959	-0.058	0.065	0.371
*CAST*									
*c.2832A>G*	BRM	0.56	0.0088	-0.113	0.065	0.082	-0.113	0.061	0.064
*c.2832A>G*	BEL	0.77	0.0195	-0.120	0.047	0.011	-0.171	0.040	0.000†
*c.2832A>G*	SGT	0.70	0.0142	-0.120	0.061	0.051	-0.140	0.055	0.011

^1^Allele frequency for allele 0, ^2 ^proportion of the residual variance explained by the SNP, ^3 ^additive effect, ^4 ^standard error of the additive effect, ^5 ^P value of the additive effect, ^6 ^allele substitution effect, ^7 ^BRM Brahman, ^8 ^BEL Belmont Red, ^9 ^SGT Santa Gertrudis, † *P *< 0.0001

Significant associations were found for haplotypes of *CAPN3 *and LLPF (Tables 5 and 6). Interval 2 (Int2), between *c.1538+225G>T *and *c.2443-103G>C*, showed significant additive or allele substitution effects in all breeds, where the haplotype substitution effect in Brahman equalled 0.22 σ_p_. The effect of Int1, between *c.53T>G *and *c.1538+225G>T*, was not significant in all breeds, and the effect of Int3, between *c.53T>G *and *c.2443-103G>C*, was not significant in any breed. Notably, the effects of Int1 and Int2 were significant in the Santa Gertrudis even though the individual locus effects were not significant in that breed. The haplotype substitution effects had more similar sizes than the individual SNP allele substitution effects in the different breeds. The haplotype substitution effect had the opposite polarity in the Santa Gertrudis to the other breeds.

**Table 5:** Means and standard errors of *CAPN3 *two-locus haplotypes and meat tenderness in different breeds

Locus	Breed	No copies^1^			One copy^2^			Two copies^3^		
		N^4^	x^5^kg	SE^6^	N	× kg	SE	N	× kg	SE
		No GT haplotypes			One GT haplotype			Two GT haplotypes		
Int1^7^	BRM^10^	185	0.13	0.06	192	-0.04	0.06	28	-0.10	0.15
Int1	BEL^11^	28	0.14	0.14	261	0.05	0.05	458	-0.02	0.03
Int1	SGT^12^	51	-0.06	0.08	282	-0.07	0.04	206	0.12	0.06
		No TG haplotypes			One TG haplotype			Two TG haplotypes		
Int2^8^	BRM	176	0.13	0.07	184	-0.01	0.06	27	-0.23	0.15
Int2	BEL	22	0.23	0.17	212	0.03	0.05	379	-0.01	0.04
Int2	SGT	49	-0.10	0.07	227	-0.07	0.05	211	0.10	0.05
		No GG haplotypes			One GG haplotype			Two GG haplotypes		
Int3^9^	BRM	61	-0.02	0.10	13	-0.29	0.24	276	0.09	0.05
Int3	BEL	24	0.10	0.13	16	0.09	0.15	467	-0.03	0.04
Int3	SGT	22	0.05	0.14	26	-0.08	0.12	354	0.05	0.04

^1^No copies of the haplotype for the common allele, ^2 ^one copy of the haplotype for the common allele, ^3 ^two copies of the haplotype of the common allele, ^4 ^N sample size, ^5 ^× average meat tenderness, ^6 ^SE standard error, ^7 ^Int1 haplotype between *CAPN3:c.53T>G *and *CAPN3:c.1538+225G>T*, ^8 ^Int2 haplotype between *CAPN3:c.1538+225G>T *and *CAPN3:c.2443-103G>C*, ^9 ^Int3 haplotype between *CAPN3:c.53T>G *and *CAPN3:c.2443-103G>C*, ^10 ^BRM Brahman, ^11 ^BEL Belmont Red, ^12 ^SGT Santa Gertrudis

**Table 6:** Association between *CAPN3 *two-locus haplotypes and meat tenderness

Locus	Breed	p_2_^1^	V_r_^2^	*a*^3 ^kg	SE^4^	P_*a*_^5^	α^6^gg	SE	P_α_
Int1^7^	BRM	0.31	0.0118	0.117	0.060	0.054	0.137	0.058	0.018
Int1	BEL	0.79	0.0031	0.077	0.046	0.094	0.073	0.042	0.082
Int1	SGT	0.64	0.0150	-0.090	0.055	0.104	-0.119	0.053	0.026
Int2^8^	BRM	0.31	0.0149	0.183	0.060	0.003	0.169	0.056	0.003
Int2	BEL	0.79	0.0040	0.122	0.044	0.005	0.076	0.042	0.069
Int2	SGT	0.67	0.0136	-0.103	0.052	0.050	-0.125	0.050	0.012
Int3^9^	BRM	0.81	0.0092	-0.057	0.092	0.540	-0.260	0.168	0.122
Int3	BEL	0.94	0.0020	0.064	0.060	0.288	0.110	0.125	0.381
Int3	SGT	0.91	0.0016	-0.001	0.081	0.992	-0.107	0.175	0.542

^1^Frequency of common haplotype, ^2 ^proportion of the residual variance explained by the common haplotype, ^3 ^additive effect, ^4 ^standard error of the additive effect, ^5 ^P value of the additive effect, ^6 ^haplotype substitution effect, ^7 ^Int1 haplotype between *CAPN3:c.53T>G *and *CAPN3:c.1538+225G>T*, ^8 ^Int2 haplotype between *CAPN3:c.1538+225G>T *and *CAPN3:c.2443-103G>C*, ^9 ^Int3 haplotype between *CAPN3:c.53T>G *and *CAPN3:c.2443-103G>C*, ^10 ^BRM Brahman, ^11 ^BEL Belmont Red, ^12 ^SGT Santa Gertrudis

The significant associations for *CAST:c2832G>A *were included because breed specific values have not previously been reported for this data set, only values for combined data (Table 4). The allele substitution effects had the same polarity in all breeds. This *CAST *SNP had similar sized effects in all three breeds. That differs from the *CAPN3 *SNP in this study, which showed different sizes in different breeds. In the Brahman, the *CAPN3:c.1538+225G>T *SNP showed a similar size to the *CAST:c.2832G>A *SNP.

We found no strong evidence of epistatic interactions between *CAPN3 *SNP and *CAST*. One comparison out of 12 had a *P *value < 0.05, for the additive × additive component in the Santa Gertrudis breed for the comparison between *CAPN3:c.1538+225G>T *and *CAST:c.2832A>G*, with *aa *= 0.19 kg, SE = 0.100, *P *value = 0.038, which was close to expected for the number of tests performed.

## Discussion

SNP at the *CAPN3 *gene confirm that there is a QTL affecting meat tenderness on bovine chromosome 10. The associations are consistent with *CAPN3 *being a positional candidate gene for the effect, with the strongest evidence found in Brahman animals. Further genotyping should be performed on neighbouring genes and the surrounding genetic region to exclude other genes. The intronic *CAPN3:c.1538+225G>T *SNP shows the largest effect, while the other SNP have weaker and inconsistent effects in different breeds. As *c.1538+225G>T *is located between the other two SNP, this might suggest that the causal mutations lie near to it. However, given the pattern of LD between the three SNP, that the two flanking SNP are in high LD in all breeds but in low LD to the central SNP in the composite breeds, it is possible that the causative mutations do not lie centrally in the gene; only the Brahman breed had some LD between *c.1538+225G>T *and the other SNP, and only in the Brahman breed was there similarity in the association to meat tenderness for all SNP. This suggests that haplotypes in the Brahman are more closely aligned to the causative mutation than in the composite breeds. Nevertheless, the differences in association between these SNP in different breeds could point to there being more than one causative mutation. These results suggest that it may be useful to discover other SNP in and around the *CAPN3 *gene and to examine these for functional, mechanistic explanations for the differences in meat tenderness as well as other traits.

The haplotype substitution effect is significant in all breeds for a haplotype that spans the 3' half of the gene. The common allele at each of the three SNP is not associated consistently with increased meat tenderness in any breed, so testing a haplotype of the common alleles is not biased towards a particular result. The analysis of the haplotypes shows stronger effects than the individual SNP associations. The significant haplotype effects suggest that a QTL exists for this trait in all breeds, despite the lack of association in the Santa Gertrudis in the single SNP analysis. The failure of the single SNP analysis in the Santa Gertrudis, as well as the haplotype substitution effect being of opposite polarity to the other breeds, suggests that the Santa Gertrudis breed has a more complex LD structure of SNP around the causative mutation. This may be a consequence of its composite origins. The failure to find evidence in any particular single SNP association test is likely to be due to differences in the amount and polarity of allelic association between the SNP and the causative mutation in that particular breed, as well as the power of the experiment to detect the association at a particular significance threshold. This echos the critique of using TAG SNP [26] as a tool for association mapping because the LD relationships between genotyped SNP cannot be used to predict the LD relationships for unknown SNP and hence for SNP-trait associations [27]. Nevertheless, the high LD between *c.53T>G *and *c.2443-103G>C *in all breeds but the failure to detect a haplotype substitution effect for these two SNP may suggest that the QTL is not in strong LD with these two SNP.

These SNP appear to be detecting a nearby QTL but they are not merely markers of difference between taurine and zebu cattle. Due to the many differences between zebu and taurine cattle, the analysis of fixed zebu-taurine differences in composite cattle is a way of tracking the influence of particular DNA segments, but such differences only explain increased genetic variability of recently crossbred cattle. However, in this case, because these DNA variants are SNP discovered in the Brahman, and are studied within that breed, the association indicated that the QTL contributes to the greater genetic variance for meat tenderness in the Brahman and other tropically adapted cattle. Identifying more SNP within the Brahman in this genetic region, particularly those in high LD to *c.1538+225G>T*, would be the start of a better understanding of how this gene or the surrounding genetic region affects meat tenderness.

## Conclusion

SNP in the positional candidate gene *CAPN3 *in a population association study confirm the location of a QTL for meat tenderness to bovine chromosome 10 originally identified in a QTL linkage experiment. The SNP variants were largely found in the zebu and composite breeds and this study implicates the QTL they detect in the greater heritability and mean trait values for meat tenderness found in zebu cattle compared to taurine cattle.

## Authors' contributions

WB planned the experiment, designed primers, analysed data and drafted the manuscript. BH and RB generated and scored genotypes for *CAPN3*. MT sequenced the *CAPN3 *gene. All authors read the manuscript.
